# Bradycardia and Heart Rate Fluctuation Are Associated with a Prolonged Intensive Care Unit Stay in Patients with Severe COVID-19

**DOI:** 10.3390/medicina58070950

**Published:** 2022-07-19

**Authors:** Pattraporn Tajarernmuang, Konlawij Trongtrakul, Warawut Chaiwong, Teerapat Nantsupawat, Athavudh Deesomchok, Panida Chanayat, Nutchanok Niyatiwatchanchai, Theerakorn Theerakittikul, Atikun Limsukon, Chaicharn Pothirat, Chalerm Liwsrisakun, Chaiwat Bumroongkit

**Affiliations:** 1 Division of Pulmonary, Critical Care, and Allergy, Department of Internal Medicine, Faculty of Medicine, Chiang Mai University, Chiang Mai 50200, Thailand; pattraporn.t@cmu.ac.th (P.T.); warawut.chai@cmu.ac.th (W.C.); athavudh.d@cmu.ac.th (A.D.); panida.kanjanavaha@cmu.ac.th (P.C.); nutchanok.n@cmu.ac.th (N.N.); theerakorn.t@cmu.ac.th (T.T.); atikun.limsukon@cmu.ac.th (A.L.); chaicharn.p@cmu.ac.th (C.P.); chalerm.liw@cmu.ac.th (C.L.); chaiwat.b@cmu.ac.th (C.B.); 2 Division of Cardiology, Department of Internal Medicine, Faculty of Medicine, Chiang Mai University, Chiang Mai 50200, Thailand; teerapat.nant@cmu.ac.th

**Keywords:** bradycardia, heart rate fluctuation, COVID-19, SARS-CoV-2

## Abstract

*Background and Objective*: Bradycardia has been observed among patients infected with severe acute respiratory syndrome coronavirus 2 (SARS-CoV-2) and is suspected to be associated with poorer outcomes. Heart rate (HR) fluctuation has been found to be correlated with a greater mortality rate in critically ill patients. The association of bradycardia and HR fluctuation with the outcome of severe coronavirus disease 2019 (COVID-19) patients has not been clarified. Therefore, we aimed to examine whether bradycardia and HR fluctuation correlated with poor outcomes in patients with severe COVID-19. *Materials and Methods:* We conducted a secondary analysis from a prospective data collection of patients admitted to the intensive care unit, between April and June 2021, at Chiang Mai University Hospital. *Results:* The results showed that 62 of 86 patients (72.1%) had bradycardia, defined by HR < 60 beats per minute (bpm). The number of patients with high HR fluctuation, defined as the difference in HR during admission ≥ 40 bpm, was greater among the bradycardia group than in the non-bradycardia group (70.9% vs. 14.7%, *p* = 0.015, respectively). The patients with bradycardia had greater levels of erythrocyte sedimentation rate (ESR) and C-reactive protein (CRP). In addition, a greater proportion of patients with bradycardia received interleukin-6 inhibitors and hemoperfusion as a rescue therapy than those with non-bradycardia. After adjusting for age, gender, body mass index, CRP, and mechanical ventilator; bradycardia and the high HR fluctuation were significantly associated with a longer length of stay in the intensive care unit (ICU-LOS), with adjusted risk ratios of 2.67, 95% CI; 1.02, 6.94, *p* = 0.045 and 2.88, 95% CI; 1.22, 6.78, *p* = 0.016, respectively. *Conclusion:* We found that bradycardia and a high heart rate fluctuation were associated with a poorer ICU outcome in terms of longer ICU-LOS among the patients with severe COVID-19.

## 1. Introduction

The emergence of the coronavirus disease 2019 (COVID-19) pandemic has led to a global health crisis, with 500 million confirmed cases and approximately 6 million deaths by early 2022 [1]. Severe acute respiratory syndrome coronavirus 2 (SARS-CoV-2) mainly attacks the respiratory tract, causing a wide range of symptoms from mild conditions, such as fever, cough, and fatigue; to acute hypoxic respiratory failure, which is the most serious complication. However, cardiac manifestations of COVID-19, including cardiomyopathy, arrhythmia, myocardial infarction, myocarditis, and coronary thromboembolism have been reported [2]. Several publications demonstrated an incidence of cardiac injury of 20% with a greater mortality rate when compared to those without cardiac involvement [3,4]. A retrospective study of 138 hospitalized patients from Wuhan, China reported an overall incidence of arrhythmia of 16.7%: affecting 44.4% of intensive care unit (ICU) patients vs. 6.9% of non-ICU patients [5].

Bradycardia in patients with COVID-19 was observed in several case series and some postulated the association with cytokine release syndrome [6,7,8,9,10,11,12]. The exact mechanism of bradycardia in COVID-19 remains unclear. Taking into consideration the existing heart rhythm alteration during COVID-19, it appears necessary to distinguish relative bradycardia from absolute bradycardia. In some studies, the presence of relative bradycardia did not seem to be associated with a worse prognosis [13]; on the other hand, the presence of absolute bradycardia may be an indication of a more severe infection. One study from the USA demonstrated higher mortality rates in patients with bradycardia than those without bradycardia [14]. In addition, a fluctuation of heart rate (HR) has been observed in many patients who were admitted to our ICU. However, there is no evidence for the correlation between HR fluctuation and worse outcomes in COVID-19 patients.

Therefore, we sought to examine whether bradycardia and high HR fluctuation were predictors of worse outcomes in patients with severe pneumonia from COVID-19. The primary outcome was an ICU length of stay (ICU-LOS) ≥ 7 days. The secondary outcomes included mortality rate and the duration of mechanical ventilator support.

## 2. Materials and Methods

### 2.1. Study Design

We conducted a secondary analysis from a prospective data collection in adult patients admitted to the ICU for emerging infectious disease (ICU-EID) at Chiang Mai University Hospital between April 2021 and June 2021. This study was approved by the Research Ethics Committee, Faculty of Medicine, Chiang Mai University (Study code: MED-2564-08109, date of approval: 3 May 2021) and filed under the Clinical Trials Registry (Study ID: TCTR20210827005, date of approval: 27 August 2021) in compliance with the Declaration of Helsinki. Written informed consent was waived owing to a state of secondary data analysis.

Data were obtained from electronic medical records of the adult patients who were diagnosed with severe COVID-19 pneumonia and admitted to the ICU-EID. COVID-19 infection was determined by the detection of SARS-CoV-2 by reverse transcriptase polymerase chain reaction (RT-PCR) from nasopharyngeal specimens. Pneumonia was diagnosed when the patients had pulmonary infiltration on chest imaging and had clinical signs and symptoms that were compatible with lower respiratory tract infection, such as fever, cough, dyspnea, and desaturation. In addition, severe pneumonia was defined when the patient was hospitalized and utilized at least one of the following: high flow nasal cannula (HFNC), non-invasive ventilation (NIV), invasive mechanical ventilation (IMV), vasopressors, dialysis, or extracorporeal membrane oxygenation (ECMO), according to the WHO Working Group on the Clinical Characterization and Management of COVID-19 infection [15]. We excluded the patients who previously received beta-blockers, digoxin, or antiarrhythmic drugs including amiodarone, flecainide, and propafenone, among others. Patients with a known history of tachy- or bradyarrhythmia were excluded from this study as well.

### 2.2. Data Collection

Patients’ demographics, pre-existing comorbidities, and duration of illness (DOI) at the onset of ICU admission were reviewed. Vital signs including HR, body temperature (BT), systolic blood pressure (SBP), diastolic blood pressure (DBP), mean arterial pressure (MAP), respiratory rate (RR), and oxygen saturation from pulse oximetry (SpO_2_) were collected at the time of ICU admission and every day at 8 AM through the ICU admission. Also, HR, RR, and SpO2 were collected every 4 h. The ROX index (SpO_2_-to-FiO_2_ ratio divided with RR) was also calculated at every instance of vital signs collection. Chest radiography severity score (chest X-ray score) was recorded at the ICU admission. The calculated chest X-ray score was defined according to the previous report [16].

The severity of illness including Acute Physiology and Chronic Health Evaluation-II (APACHE-II) score, Sequential Organ Failure Assessment Score (SOFA) score, and National Early Weaning Score (NEWS) was also collected. Laboratory data including complete blood count (CBC); blood chemistry, inflammatory markers including erythrocyte sedimentation rate (ESR) and C-reactive protein (CRP); treatment information (respiratory support and medications); and outcomes including duration of high flow nasal cannula (HFNC), duration of mechanical ventilator use, ICU-LOS, and mortality rate were also recorded. Twelve-lead electrocardiography (ECG) was performed in patients whose central monitor ECG showed abnormal rhythm other than sinus bradycardia.

### 2.3. Definitions

Bradycardia and significant bradycardia were defined as the patients’ HR < 60 beats per minute (bpm) and <50 bpm for at least two consecutive occasions, respectively. A low HR before death was not counted. A prolonged ICU-LOS was defined as the requirement of ICU admission ≥ seven days. The HR fluctuation was calculated according to a difference between maximum HR (HR_max_) and minimum HR (HR_min_) during the ICU admission. High HR fluctuation was defined when this difference was ≥ 40 bpm.

### 2.4. Sample Size Estimation

The sample size was estimated from the study by Kumar S, et al. [14]. Using the adjusted odds ratio of 6.59, the proportion of death in subjects with bradycardia and normal HR was 23.4% and 5.1%, respectively. Therefore, 81 subjects (54 bradycardia) were needed to be involved to reject the null hypothesis with the power of 0.8 and the assumption of statistical significance level at 0.05.

### 2.5. Statistical Analysis

Categorical data were expressed as numbers and percentages, while continuous data were expressed using the median and interquartile. A comparison of categorical variables between groups was analyzed using Fisher’s exact test. A comparison of continuous variables was performed using a Mann-Whitney U test. The generalized linear model was performed to identify the bradycardia and the HR fluctuation during admission (HR_max_ − HR_min_) as a predictor for an ICU-LOS ≥ seven days, with an adjustment for possible confounding factors including pre-treatment factors, laboratory results, and post-treatment factors. These included age, sex, body mass index (BMI), C-reactive protein (CRP), and mechanical ventilator use. The results were displayed as adjusted risk ratio, together with a 95% confidence interval (95% CI). A *p*-value < 0.05 was considered statistically significant. All statistical analyses were performed using STATA version 16 (StataCorp, College Station, TX, USA).

## 3. Results

A total of 86 patients with severe COVID-19 pneumonia were involved. Of these, 62 patients (72.1%) had bradycardia (HR < 60 bpm). Among the patients with bradycardia, 20/62 (32.2%) patients experienced significant bradycardia (HR < 50 bpm) during admission. There were no significant differences in baseline characteristics between the two groups (Table 1). We found two patients with co-existing heart disease; however, they had no history of abnormal HR and did not formerly receive beta-blockers. The bradycardia group had a lower mean HR and a higher HR fluctuation during admission than the non-bradycardia group. Nonetheless, no advanced atrioventricular (AV) block was observed in our study.

The patients with bradycardia had significantly higher initial ESR levels, 32.5 (IQR 24.3, 51.0) mm/h vs. 24.5 (IQR 7.3, 24.5) mm/h, *p* = 0.028. The initial CRP level was not different between the two groups (Table 2); however, the peak CRP levels in some patients were observed a few days later after admission, with a maximum value of CRP level at 111.4 (IQR 62.6, 172.6) mg/L vs. 68.6 (IQR 41.9, 126.7) mg/L, respectively, *p* = 0.011.

Interestingly, we found an initial ROX index that was lower in the patients with bradycardia (Table 2). Likewise, the median value of the lowest ROX index during admission trended toward lower in the bradycardia group compared to those in the non-bradycardia group; 7.2 (IQR 4.6, 8.2) vs. 8.3 (IQR 6.7, 9.6), *p* = 0.051.

Clinical outcomes including the duration of HFNC use and ICU-LOS were significantly longer in the patients with bradycardia. The median duration of HFNC usage in patients with bradycardia was 5.0 (IQR 3.3, 6.0) days, while it was 4.0 (IQR 3.0, 4.5) days in those with non-bradycardia, *p* = 0.032. The median duration of ICU-LOS in the bradycardia group was 8.0 (IQR 6.0, 12.0) days compared to 5.0 (IQR 4.0, 6.0) days in the non-bradycardia group, *p* < 0.001.

In addition, 14 (22.9%) patients who received hemoperfusion as a rescue treatment had bradycardia. There were seven (8.1%) patients who died, and six of them had bradycardia during admission (Table 2). All six patients in the bradycardia group died from severe COVID-19 pneumonia. On the contrary, one patient in the non-bradycardia group died from acute limb ischemia with reperfusion syndrome without evidence of severe pneumonia.

**Figure 1:**
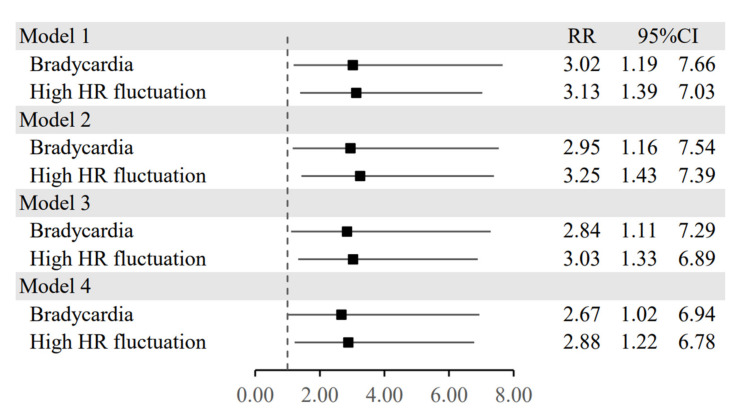
Risk ratio plots for univariable (Model 1) and multivariable regression analyses adjusted for covariates (Models 2–4) of the patients with bradycardia and high heart rate fluctuation as predictors of a prolonged ICU stay. Note: Model 1: Univariable analysis; Model 2: Multivariable analysis adjusted for pre-treatment covariates including age, sex, and BMI; Model 3: Multivariable analysis adjusted for pre-treatment covariates and laboratory results including age, sex, BMI, and CRP; Model 4: Multivariable analysis adjusted for pre-treatment covariates, laboratory results, and post-treatment including age, sex, BMI, CRP, and mechanical ventilator usage.

The generalized linear model showed that patients with bradycardia had significantly longer ICU-LOS, with a crude risk ratio of 3.02; 95%CI, 1.19–7.66; *p* = 0.020 in model 1. This significance remained when adjusted with other covariates in models 2–4 (Table S1). In addition, there was also a significant association between a high HR fluctuation (HR_max_ − HR_min_ ≥ 40 bpm) and a prolonged ICU-LOS, with a crude risk ratio of 3.13; 95%CI, 1.39–7.03, *p* = 0.006 in model 1. The adjusted risk ratio remained significant when adjusted with other covariates in models 2–4 (Table S2). The risk plot of each model for bradycardia and a high HR fluctuation affecting ICU-LOS was demonstrated in Figure 1.

## 4. Discussion

Cardiac manifestation has been frequently observed in severe COVID-19 infection. A previous study showed that sinus bradycardia was observed in 15% of moderate COVID-19 patients [17]. This study also demonstrated that patients did not have any signs of cardiomyopathy, and subsequently resolved when the clinical condition of COVID-19 was improved [17]. In the literature, several cases of heart rhythm disturbances have been described during SARS-CoV-2 infection, requiring, in some cases, definitive pacemaker positioning [18]. In these cases, the implantation of a loop recorder with a remote monitoring may represent a valid tool for the follow up of patients experiencing severe bradycardia during or as a consequence of SARS-CoV-2 infection [19,20]. Kumar S, et al. reported an incidence of absolute bradycardia (HR < 60 bpm) of 24.9% in COVID-19 patients. The overall mortality was 18.7%, while a higher mortality rate was observed among patients with bradycardia than in the non-bradycardia group (17.6% vs. 4.6%) [14].

This is the first study on the incidence of bradycardia and the outcomes among severe and critically ill COVID-19 patients. A high incidence of bradycardia (72.1%) was observed among patients admitted to the ICU. A poorer outcome in terms of a significantly longer ICU-LOS, longer HFNC use, and more frequent use of rescue therapies including interleukin-6 (IL-6) receptor inhibitors and hemoperfusion was observed in the bradycardia group. The mortality rate was not significantly greater in the bradycardia group (9.7% vs. 4.7%, *p* = 0.668). However, a previous report demonstrated that bradycardia in critically ill COVID-19 patients (SARS-CoV-2 B.1.1.7 Lineage) was associated with significantly greater mortality (64% vs. 11%, *p* < 0.001) [21]. Moreover, this study also exhibited a significantly higher level of urokinase plasminogen activator receptor (suPAR) levels in patients with bradycardia than in those without bradycardia (9.07 ± 4.3 ng/mL vs. 7.65 ± 5.2 ng/mL, respectively, *p* < 0.001) [21].

Interestingly, the patients with bradycardia had a higher level of inflammatory markers including ESR and CRP. Thus, bradycardia is likely to be an indicator of a high severity of the disease and possibly a warning sign of a cytokines storm. Although IL-6 levels were not routinely measured in our study, a previous study demonstrated that IL-6 can increase the level of vagal tone stimulus and decrease HR variability [22]. Therefore, the use of the IL-6 receptor inhibitor can suppress the effect of cytokines on HR variation [22].

The dissociation between pulse and temperature called relative bradycardia has been reported in several infectious diseases such as typhoid fever, legionella, and malaria [23]. Lately, bradycardia has also been found in COVID-19 infection. The pathogenesis of bradycardia in COVID-19 infection remains unclear. Several mechanisms of bradyarrhythmia in COVID-19 infection have been proposed. First, the most accepted mechanism in myocardial damage is a direct invasion of the virus to the sinoatrial (SA) nodal cells where the angiotensin-converting enzyme-2 (ACE-2) receptors, where SARS-CoV-2 virus enters the host cells, are located [24]. Second, an elevation of several cytokines during an overwhelming immunologic response can alter the expression and function of calcium and potassium channels on the cardiac cell membrane, causing the alteration of HR response [22]. Increasing the blood level of circulating IL-6 is significantly correlated with a depression of HR variability in an animal model [25]. Third, the presence of intracellular virus in myocardial cells stimulates macrophage migration and cytotoxic T-cells, causing myocarditis [26]. Additionally, other triggers for arrhythmia include electrolyte abnormalities, hypoxemia, myocardial ischemia, myocardial strain, or side effects from medications [27]. Attena E, et al. reported the presence of sinus bradycardia following the administration of intravenous remdesivir for the treatment of COVID-19 [28]. However, evidence of acute myocardial ischemia and severe electrolyte derangement were not observed in our study. Besides, the proportion of patients who received remdesivir in the present study did not differ between the two groups. We believed that bradycardia was more likely to be associated with the SARS-CoV-2 viral infection rather than the administration of remdesivir.

Our study also found that the patients with bradycardia had high HR fluctuation. One study demonstrated a higher risk of 28-day and one-year mortality in critically ill ICU patients with high HR fluctuations of more than or equal to 35 bpm [29]. After adjusting for other confounders, the high HR fluctuation was found to be associated with a longer ICU-LOS. The mechanism of high HR fluctuation is unclear. We hypothesize that the multiple factors discussed above cause autonomic nervous system disturbance and an impaired circadian change in HR.

## 5. Limitations

This study had the main limitation of being a single center study. The patients were admitted to the ICU at a median time of four to five days after the DOI. The data on inflammatory cytokines and HR before admission was lacking, so we could not demonstrate the level of inflammation and the onset of bradycardia. Furthermore, many patients with bradycardia received IL-6 inhibitors and hemoperfusion as rescue therapy. Generally, the use of IL-6 inhibitors could suppress the expression of IL-6, resulting in an attenuation of an increase in vagal tone and a decrease in HR variability [22]. However, IL-6 was not measured in our study. Therefore, the data was insufficient to conclude how IL-6 inhibitors affect bradycardia. Also, the patients who were intubated might have been given sedative drugs and opioids such as dexmedetomidine, propofol, or fentanyl that would significantly interfere with their HR [30,31]. Unfortunately, this information was not obtained in our study. In addition, HR variability and signs of autonomic dysfunction measurement were not studied. Therefore, studies to prove how autonomic dysfunction is associated with bradycardia or HR fluctuation in patients with COVID-19 pneumonia should be investigated further. Next, the timing of HR fluctuation was calculated from a difference between maximum- and minimum-value during the patients’ ICU admission. This time frame for observation might be too long. Also, HR fluctuation might be affected by other para-physiological conditions such as the presence of fever or pain. Apart from HR, the detailed analysis of a simple instrument such as a 12-lead ECG may provide useful prognostic information [32,33]. Lastly, we did not collect long-term cardiac sequelae in the survivors. Future studies are needed.

## 6. Conclusions

The incidence of bradycardia in severe and critically ill COVID-19 patients was high and was associated with poorer outcomes when compared to those without bradycardia. Additionally, a high HR fluctuation also indicated poor outcomes in terms of longer ICU-LOS. This could be an indicator of autonomic dysfunction or an impaired circadian rhythm. However, further investigation is needed to investigate this phenomenon.

## Figures and Tables

**Table 1:** Demographic data of COVID-19 pneumonia subjects (N = 86).

Variables	Bradycardia (N = 62)	No Bradycardia (N = 24)	*p*-Value
Demographic data			
Age (year)	52.0 (35.0, 62.0)	47.0 (35.0, 61.7)	0.655
Male, *n* (%)	33 (53.2)	15 (62.5)	0.477
Body weight (kg)	76.0 (63.0, 94.0)	76.0 (66.0, 91.0)	0.900
Body mass index (kg/m^2^)	28.1 (24.0, 34.8)	27.7 (25.8, 31.2)	0.947
Lists of co-morbidities, *n* (%)			
Heart diseases	1 (1.6)	1 (4.2)	>0.999
Diabetes mellitus	9 (14.5)	2 (12.5)	>0.999
Chronic Kidney disease	3 (4.8)	0 (0.0)	0.557
Hypertension	32 (52.5)	12 (50.0)	>0.999
Symptoms, *n* (%)			
Fever	46 (74.2)	17 (70.8)	0.789
Cough	45 (72.6)	18 (75.0)	>0.999
Dyspnea	38 (61.3)	16 (66.7)	0.804
Muscle pain	11 (17.7)	3 (12.5)	0.748
Diarrhea	17 (27.4)	7 (29.2)	>0.999
Anosmia	4 (6.5)	0 (0.0)	0.573

Data are presented as median and interquartile or *n* (%).

**Table 2:** Data during ICU admission of COVID-19 pneumonia (N = 86).

Variables	Bradycardia (N = 62)	No Bradycardia (N = 24)	*p*-Value
Duration of illness before hospitalization (days)	5.0 (3.0, 7.3)	4.0 (2.3, 7.0)	0.507
Vital Signs at ICU Admission			
Body temperature (°C)	37.6 (36.7, 38.5)	37.2 (36.8, 38.3)	0.623
Heart rate (bpm)	91.0 (81.0, 100.0)	92.0 (80.0, 100.0)	0.452
Respiratory rate (breath/min)	24.0 (20.0, 26.0)	24.0 (20.0, 27.0)	0.992
Systolic blood pressure (mmHg)	126.0 (112.0, 141.0)	130.0 (118.0, 140.0)	0.328
Diastolic blood pressure (mmHg)	78.0 (65.0, 86.0)	78.0 (70.0, 88.0)	0.333
Mean arterial pressure (mmHg)	92 (81.0, 102.0)	94 (91.0, 105.0)	0.306
Oxygen saturation (%)	92.0 (89.0, 96.0)	94.0 (90.0, 96.0)	0.341
Heart Rate Data During Admission			
Minimum HR during admission (bpm)	50.0 (48.0, 55.0)	60.0 (60.0, 68.0)	<0.001
Maximum HR during admission (bpm)	100.0 (94.0, 110.0)	100.0 (86.0, 112.0)	0.876
HR_max_ − HR_min_ during admission (bpm)	50.0 (38.0, 62.0)	34.0 (24.0, 48.0)	<0.001
High HR fluctuation (≥40 bpm), *n* (%)	44 (70.9)	10 (14.7)	0.015
Inflammatory Biomarkers C-reactive protein (mg/L) Erythrocyte sedimetation rate (mm/hr) D-dimer (ng/mL) Hematological and Chemistry Data	88.6 (8.9, 130.1) 32.5 (24.3, 51.0) 490.0 (363.0, 1129.0) 0.14 (0.08, 0.44)	68.8 (41.9, 126.7) 24.5 (7.3, 24.5) 493.0 (385.5, 965.0) 0.15 (0.08, 0.36)	0.416 0.028 0.956 0.936
Hemoglobin (g/dL)	13.9 (12.4, 14.8)	13.0 (11.8, 14.4)	0.229
Hematocrit (%)	40.2 (35.8, 43.3)	39.7 (33.9, 44.1)	0.767
White blood cells (×10^3^ cells/mm^3^)	6.7 (4.8, 9.8)	5.3 (4.7, 7.5)	0.129
Neutrophil (×10^3^ cells/mm^3^)	6.3 (4.0, 8.1)	5.4 (3.9, 7.8)	0.269
Lymphocyte (×10^3^ cells/mm^3^)	1.1 (0.9, 1.5)	1.3 (0.6, 2.0)	0.576
Platelet (×10^3^ cells/mm^3^)	197.0 (149.0, 273.0)	207.0 (170.0, 290.0)	0.659
Blood urea nitrogen (mg/dL)	14.0 (11.0, 19.0)	14.0 (11.0, 16.0)	0.750
Creatinine (mg/dL)	0.91 (0.73, 1.08)	0.93 (0.71, 1.09)	0.945
Sodium (mmol/L)	136.0 (133.0, 138.0)	136.5 (133.5, 138.5)	0.797
Potassium (mmol/L)	3.8 (3.4, 4.1)	3.7 (3.2, 4.2)	0.543
CXR score at admission	14.0 (9.0, 19.0)	16.0 (9.0, 21.0)	0.329
APACHE-II score	8.0 (3.0, 13.0)	9.0 (4.5, 12.0)	0.779
SOFA score	2.0 (2.0, 3.0)	2.0 (2.0, 3.0)	0.857
NEWS score	5.0 (3.0, 7.0)	4.5 (2.0, 6.0)	0.320
ROX index at admission	9.5 (6.7, 14.0)	11.2 (8.6, 15.8)	0.195
Management at ICU Admission			
Remdesivir, *n* (%)	39 (62.9%)	12 (50.0%)	0.331
Favipiravir, *n* (%)	23 (37.1%)	12 (50.0%)	0.331
Systemic corticosteroid, *n* (%)	55 (88.7)	21 (87.5)	1.000
Vasopressor, *n* (%)	9 (14.5)	1 (4.7)	0.271
Interleukin-6 inhibitor, *n* (%)	23 (37.1)	1 (4.2)	0.002
Hemoperfusion, *n* (%)	14 (22.9)	0 (0.0)	0.009
HFNC usage, *n* (%)	48 (77.4)	18 (75.0)	0.784
HFNC usage (days) (*n* = 66)	5.0 (3.3, 6.0)	4.0 (3.0, 4.5)	0.032
Mechanical ventilator usage, *n* (%)	14 (22.6)	1 (4.2)	0.057
Mechanical ventilator usage (days) (*n* = 15)	9.0 (7.0, 14.5)	2.0 (2.0, 2.0)	0.133
ICU length of stay (days)	8.0 (6.0, 12.0)	5.0 (4.0, 6.0)	<0.001
ICU mortality rate, *n* (%)	6 (9.7)	1 (4.7)	0.668

Data are presented as median and interquartile or *n* (%). Abbreviations: APACHE-II score, Acute Physiology and Chronic Health Evaluation-II score; CXR, chest X-ray; ICU, intensive care unit; HFNC, High flow nasal cannula; HR, heart rate; HR_max_, maximum heart rate; HR_min_, minimum heart rate; NEWS score, National Early Warning Score; SOFA score, Sequential Organ Failure Assessment score.

## Data Availability

The data presented in this study are available from the corresponding author upon reasonable request.

## Supplementary Materials

The following supporting information can be downloaded at: https://www.mdpi.com/article/10.3390/medicina58070950/s1, Table S1: Multivariable analysis identifying bradycardia as a predictor of a prolonged ICU length of stay (≥7 days) in severe COVID-19 pneumonia subjects. Table S2: Multivariable analysis identifying the high HR fluctuation during admission as a predictor of a prolonged ICU length of stay (≥7 days) in severe COVID-19 pneumonia subjects.
