# Supplementary material for: Bradycardia and Heart Rate Fluctuation Are Associated with a Prolonged Intensive Care Unit Stay in Patients with Severe COVID-19
Source: Medicina (Kaunas). 2022 Jul 19;58(7):950. doi: 10.3390/medicina58070950 (PMC9319932; doi:10.3390/medicina58070950)
Supplement: Supplementary file 1 [file medicina-58-00950-s001.zip › medicina-1798996-supplementary.pdf]

Bradycardia and Heart Rate Fluctuation are Associated with a Prolonged Intensive Care Unit Stay in Patients with Severe COVID-19

Supplement Materials

Table S1 Multivariable analysis identifying bradycardia as a predictor of a prolonged ICU length of stay (≥ 7 days) in severe COVID-19 pneumonia subjects.

Risk Factors	Risk Ratio	95% CI	p-value
Model 1			
Bradycardia	3.02	1.19, 7.66	0.020
Model 2			
Bradycardia	2.95	1.16, 7.54	0.023
age	1.00	0.98, 1.02	0.731
Male	0.86	0.46, 1.59	0.629
Body mass index	1.00	0.95, 1.05	0.980
Model 3			
Bradycardia	2.84	1.11, 7.29	0.030
age	1.00	0.98, 1.02	0.832
Male sex	0.86	0.45, 1.62	0.631
BMI	0.99	0.94, 1.05	0.889
C-reactive protein	1.00	0.99, 1.00	0.875
Model 4			
Bradycardia	2.67	1.02, 6.94	0.045
age	0.99	0.97, 1.02	0.865
Male	0.86	0.45, 1.62	0.632
Body mass index	0.99	0.94, 1.05	0.877
C-reactive protien	1.00	0.99, 1.00	0.937
Mechanical ventilator used	1.45	0.66, 3.16	0.351

Note: Model 1: Univariable analysis; Model 2: Multivariable analysis adjusted for pre-treatment covariates including age, sex, and BMI; Model 3: Multivariable analysis adjusted for pre-treatment covariates and laboratory results including age, sex, BMI, and CRP; Model 4: Multivariable analysis adjusted for pre-treatment covariates, laboratory results, and post-treatment including age, sex, BMI, CRP, and mechanical ventilator used.

Table S2 Multivariable analysis identifying the high HR fluctuation during admission as a predictor of a prolonged ICU length of stay (≥ 7 days) in severe COVID-19 pneumonia subjects.

Risk Factors	Adjusted risks ratio	95% CI	p-value
Model 1			
High HR fluctuation during admission	3.13	1.39, 7.03	0.006
Model 2			
High HR fluctuation during admission	3.25	1.43, 7.39	0.005
Age	1.00	0.99, 1.02	0.442
Male	0.93	0.50, 1.72	0.815
Body mass index	0.99	0.94, 1.05	0.940
Model 3			
High HR fluctuation during admission	3.03	1.33, 6.89	0.008
Age	1.01	0.98, 1.03	0.442
Male	0.95	0.49, 1.79	0.867
Body mass index	0.99	0.94, 1.05	0.930
C-reactive protein	1.00	0.99, 1.00	0.760
Model 4			
High HR fluctuation during admission	2.88	1.22, 6.78	0.016
Age	1.00	0.98, 1.03	0.639
Male	0.93	0.49, 1.77	0.833
Body mass index	0.99	0.94, 1.05	0.919
C-reactive protein	1.00	0.99, 1.00	0.801
Mechanical ventilator used	1.19	0.52, 2.74	0.668

Note: Model 1: Univariable analysis; Model 2: Multivariable analysis adjusted for pre-treatment covariates including age, sex, and BMI; Model 3: Multivariable analysis adjusted for pre-treatment covariates and laboratory results including age, sex, BMI, and CRP; Model 4: Multivariable analysis adjusted for pre-treatment covariates, laboratory results, and post-treatment including age, sex, BMI, CRP, and mechanical ventilator used.
